# Severe Hypertriglyceridemia due to a novel p.Q240H mutation in the Lipoprotein Lipase gene

**DOI:** 10.1186/s12944-015-0107-1

**Published:** 2015-09-04

**Authors:** Angela Ganan Soto, Adam McIntyre, Sungeeta Agrawal, Shara R. Bialo, Robert A. Hegele, Charlotte M. Boney

**Affiliations:** Department of Pediatrics, Rhode Island Hospital and Brown University, Providence, RI USA; Robarts Research Institute, Western University, London, ON Canada; Baystate Children’s Hospital, 759 Chestnut Ave S584, Springfield, MA USA

**Keywords:** Chylomicronemia, Triglyceride, Lipoprotein lipase deficiency

## Abstract

**Background:**

Lipoprotein Lipase (LPL) deficiency is a rare autosomal recessive disorder with a heterogeneous clinical presentation. Several mutations in the *LPL* gene have been identified to cause decreased activity of the enzyme.

**Findings:**

An 11-week-old, exclusively breastfed male presented with coffee-ground emesis, melena, xanthomas, lipemia retinalis and chylomicronemia. Genomic DNA analysis identified lipoprotein lipase deficiency due to compound heterozygosity including a novel p.Q240H mutation in exon 5 of the lipoprotein lipase (*LPL*) gene. His severe hypertriglyceridemia, including xanthomas, resolved with dietary long-chain fat restriction.

**Conclusions:**

We describe a novel mutation of the *LPL* gene causing severe hypertriglyceridemia and report the response to treatment. A review of the current literature regarding LPL deficiency syndrome reveals a few potential new therapies under investigation.

## Findings

Exogenous lipid metabolism involves packaging of dietary fat into chylomicrons in the small intestine, which are later transported to the bloodstream. The initial step of endogenous lipid metabolism involves the hydrolysis of chylomicrons by lipoprotein lipase (LPL) [1]. LPL deficiency is a rare autosomal recessive disorder with a prevalence of 1 in 1,000,000 in the United States and higher in other world regions, like Quebec, Canada due to a founder effect [2]. The clinical presentation can include moderate or severe hypertriglyceridemia in affected homozygotes or compound heterozygotes, while simple heterozygote carriers often have normal lipids [3]. Clinical features include eruptive xanthomas, abdominal pain, hepato-splenomegaly, lower gastrointestinal bleeding, recurrent pancreatitis, or lipemia retinalis [4]. Dietary fat restriction is the cornerstone of treatment, and gene therapy trials are ongoing [5].

### Case report

**Fig. 1:**
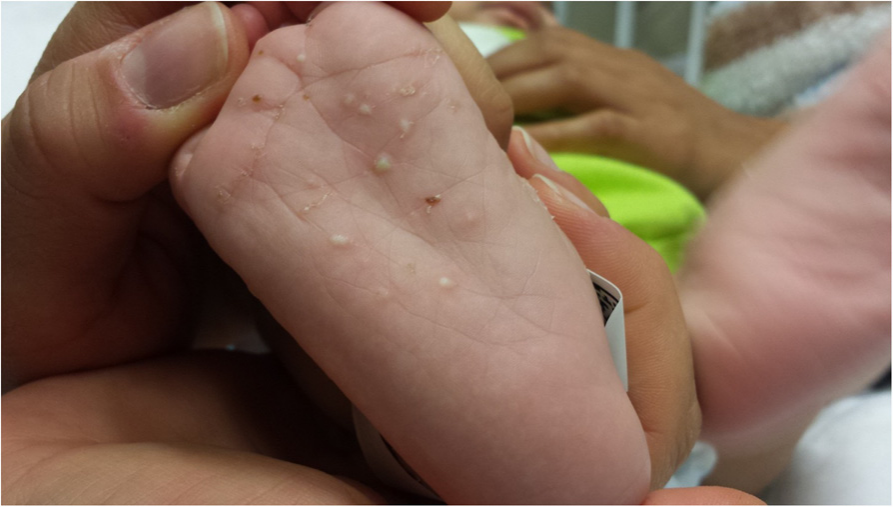
Eruptive xanthomas on the right sole

We report the case of an 11-week-old male of Puerto Rican descent who presented to the emergency department with coffee-ground emesis, melena and a papular rash. He was born full term to healthy, nonconsanguineous parents with a birth weight of 8 lb 4 oz and was exclusively breast-fed. He had 4–5 episodes of coffee-ground emesis, one episode of melena and a papular rash (Fig. 1) that spread from his extremities to his trunk over the course of 3 weeks. CBC revealed a hematocrit of 17 % and his blood was grossly lipemic. A random lipid panel showed triglycerides (TG) of 43,980 mg/dl.

The physical exam was notable for a well-appearing infant with diffuse 2 mm yellow papules consistent with eruptive xanthomas and lipemia retinalis on fundoscopic exam. Fasting lipid profile revealed total cholesterol 768 mg/dl, TG 37,695 mg/dl, and HDL < 10 mg/dl. Breast-feeding was discontinued and he was placed on Enfaport Lipil, a formula with 84 % of fat content from medium-chain triglycerides (MCT) and 16 % from long-chain fats. A repeat lipid profile one month after the formula change revealed dramatic improvement in TG (Table 1). Two months after treatment, xanthomas and lipemia retinalis resolved; weight gain and linear growth were normal. We hypothesized that this child had an autosomal recessive mutation in the *LPL* gene.

**Table 1:** Plasma lipid levels of the proband at baseline and after 1 month of dietary fat restriction, and both parents

Lipid profile	Proband			Mother	Father
	Baseline (non-fasting)	Baseline (fasting)	After dietary change		
TG (mg/dl)	43,980	37,695	375	87	78
TC (mg/dl)	761	768	98	190	127
HDL (mg/dl)	37	<10	21	43	41
LDL (mg/dl)	Unable to measure	Unable to measure	2	130	70

*TG* triglycerides, *TC* total cholesterol, *HDL* high-density lipoprotein, *LDL* low-density lipoprotein

### Methods

Ethics, consent and permission:Approval was obtained from the Western University Ethics Review Board protocol 07920E and informed consent was obtained from both parents. The parents gave their consent to publish this case.

Genomic DNA was isolated from whole blood using Puregene DNA isolation kit (Gentra Systems, QIAGEN Inc, Mississauga, ON, Canada). PCR amplifications were performed using primers covering the coding regions and ~100 bp of intron-exon boundaries of the *LPL* gene as described previously [3]. Bi-directional Sanger sequencing was performed using established conditions [3] on an ABI 3730 DNA Analyzer (Applied Biosystems, Foster City, CA, USA). DNA sequences were analyzed using SeqScape v2.6 (Applied Biosystems, Foster City, CA, USA).

### Results and discussion

**Fig. 2:**
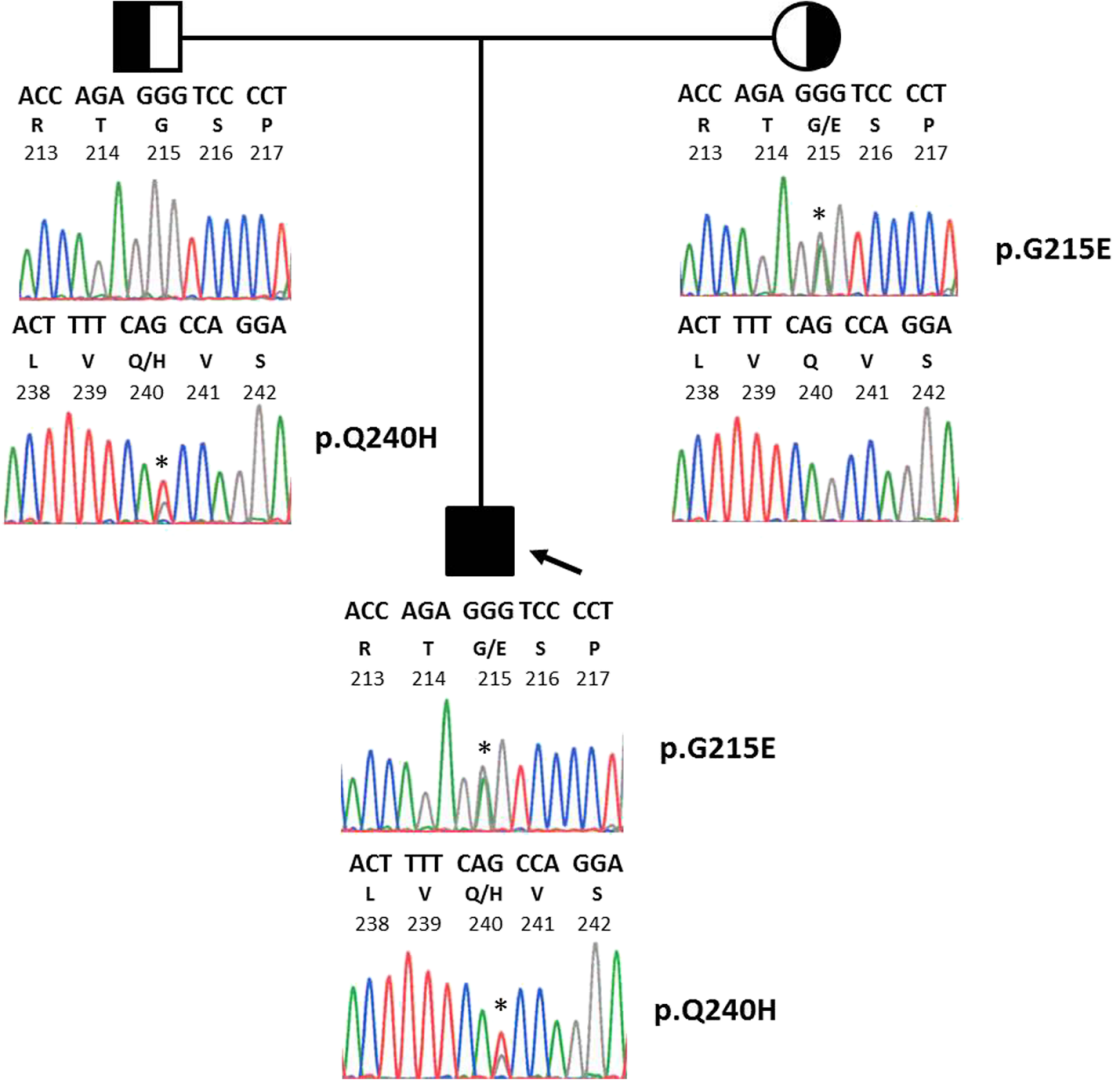
Segregation of *LPL* mutations in index family. Pedigree structure and DNA sequence electropherogram tracings of the LPL gene in the vicinity of codons 215 and 240 for each family member are shown. Three letter nucleotide sequences, single letter amino acid codes, and codon numbers corresponding to amino acid residues are indicated. Asterisks show heterozygosity at the specific amino acid position: the father is a simple heterozygote for the p.Q240H mutation, the mother is a simple heterozygote for the p.G215E mutation and the proband is a compound heterozygote for both mutations

We found that the proband was a compound heterozygote for one novel and one reported mutation within exon 5 of the *LPL* gene (Fig. 2). The first mutation p.G215E was a heterozygous transition c.644G → A, resulting in a substitution of glycine to glutamic acid at amino acid 215 (identical to residue 188 in the mature protein). This mutation has been previously reported to cause LPL deficiency [6]. The second, novel mutation p.Q240H was a heterozygous transversion c.721G → T, causing an amino acid substitution of glutamine to histidine at residue 240 (identical to residue 213 in the mature protein). In silico software programs PolyPhen2 and SIFT both predicted that this mutation was probably damaging. Both parents' DNA samples were sequenced: the LPL p.Q240H mutation was found in the father and the p.G215E mutation was found in the mother.

LPL plays a role in multiple stages of lipid metabolism, including hydrolysis of chylomicrons. LPL is primarily synthesized by myocytes and adipocytes and recent studies have identified glycosylphosphatidylinositol-anchored high-density lipoprotein-binding protein 1 (GPIHBP1) as the molecule responsible for transporting LPL to the capillary lumen [7]. LPL deficiency is a rare autosomal recessive disorder caused by decreased LPL activity due to mutations in the *LPL* gene [2]. The *LPL* gene is located on chromosome 8p21.3 and contains 10 exons, spanning ~ 30 kb and encodes a mature protein of 448 amino acids [8]. Most mutations are found in exons 4, 5 and 6 and although missense mutations are more common, nonsense, frameshift, insertion, deletion and duplication mutations have also been described [9]. The mutation in this patient expands the spectrum of known pathogenic LPL mutations to more than 150. It also occurs in a region of the protein that has to date been relatively bereft of reported mutations. The involvement of amino acid residue 240 also highlights the functional importance of the local domain in lipolysis of triglyceride-rich lipoproteins.

To date, genotype-phenotype correlations have not been identified [2, 10]. Patients who are homozygous or compound heterozygotes can present with marked hypertriglyceridemia. Heterozygote patients can have normal or mildly elevated TG levels [3]. Both parents of our patient are heterozygous carriers of *LPL* gene mutations and had normal lipids.

The clinical manifestations of LPL deficiency are heterogeneous. Eruptive xanthomas appear mostly on shoulders, buttocks and extensor surfaces of the limbs when the TG levels exceed 2000 mg/dl [11]. Lipemia retinalis involving the peripheral vessels can be seen with TG levels over 2,500 mg/dl and as the levels increase, the damage extends to the posterior pole [12]. Abdominal pain and gastrointestinal hemorrhage have been attributed to hyperviscosity secondary to elevated TG [13]. Pancreatitis is common in these patients and risk increases with TG levels over 1,000 mg/dl [14]. Therefore, the goal of therapy is to decrease TG levels < 1,000 mg/dl [15].

Dietary restriction remains the cornerstone of treatment and fat supplements using MCT are useful since they are absorbed directly into the portal vein [15]. Long-term data on patients with LPL deficiency are limited but show normal linear growth and puberty despite dietary fat restriction [4]. Even with recurrent pancreatitis, this population does not demonstrate a high mortality rate [16]. Satisfactory TG levels can be difficult to achieve with diet restriction alone. Drug therapy using gemfibrozil, omega-3 fatty acids and orlistat may decrease TG levels when combined with a fat-restricted diet however, the use of these drugs is controversial [3, 17, 18]. Therapy involving intramuscular introduction of a gain-of-function variant of the *LPL* gene is approved in Europe, although is costly and has only a transient effect [5]. A promising treatment approach currently under investigation is orally-administered diacylglycerol O-acyltransferase 1 (DGAT1) inhibitors targeting DGAT1, which mediates triglyceride synthesis during dietary fat absorption [19].

Our patient is a compound heterozygote who has a novel p.Q240H mutation in exon 5 of the *LPL* gene causing severe hypertriglyceridemia. Although his TG levels are much improved with MCT-based formula, the challenge will be maintaining those levels with dietary fat restriction as table foods are introduced and he grows through childhood.
